# Takayasu arteritis and hyperthyroidism: A secondary hypertension case report

**DOI:** 10.1097/MD.0000000000035623

**Published:** 2023-11-24

**Authors:** Lian-Man He, Min Liu, Wen-Yong Dong, Xiao-Lin Sun

**Affiliations:** a Department of Hypertension, Henan Province People’s Hospital, Zheng Zhou, China.

**Keywords:** hyperthyroidism, secondary hypertension, Takayasu arteritis

## Abstract

**Introduction:**

Renovascular disease and hyperthyroidism are secondary hypertension. Takayasu arteritis (TAK) is a chronic, progressive, nonspecific great vasculitis involving the aorta and its major branches. It is one of the causes of renal artery stenosis. Hyperthyroidism is an endocrine disease caused by improper continuous synthesis and secretion of excessive thyroid hormone by the thyroid gland. Both diseases can raise blood pressure (BP).

**Case presentation:**

we present a case of 18-year-old. Female, after exercise, fatigue palpitations. The maximum BP was 190/87 mm Hg, ankle-brachial index was <0.9. C-reactive protein and erythrocyte sedimentation rate were elevated. Imaging revealed multiple vascular stenosis. Triiodothyronine, tetraiodothyroxine, serum-free triiodothyronine, serum-free thyroxine, thyroid peroxidase antibody and thyroid stimulating receptor antibody were elevated. TSH reduced. She was diagnosed with TAK and hyperthyroidism. After treatment, the BP was normal, the thyroid function gradually returned to normal, and the symptoms improved.

**Conclusion:**

It is suggested that the BP of both upper limbs should be measured in newly diagnostic hypertension. If BP is not measured in both upper limbs, it is likely to be missed diagnosis. The cause of vascular stenosis needs to be identified, otherwise interventional treatment may lead to aggravation of the condition. Few cases of TAK complicated with hyperthyroidism have been reported. Both diseases are related to the immune system, whether there is any correlation between the 2 diseases, further research is needed. Early diagnosis, early treatment, the earlier intervention, the better prognosis.

## 1. Introduction

The prevalence of secondary hypertension is reported to be 5% to 15%, the prevalence of renovascular disease in hypertensive patients is reported to be 1% to 10%. The prevalence of Thyroid disease (hyper or hypothyroidism) in hypertensive patients is reported to be 1% to 2%.^[1]^

Takayasu arteritis (TAK) has been found worldwide and is most common in young Asian women. The age of onset was <40 years old, the ratio of male to female is about 1:4 to 9.^[2]^ The pathogenesis of TAK has not been clarified yet, but the present study found that TAK is characterized by the onset of adventitia, which can be characterized by a large number of inflammatory cells infiltration and fibrosis in the adventitia of aorta at the early stage of the disease, and gradually involve the whole layer, it leads to significant thickening, stiffness, decreased compliance of the vessel wall, stenosis or even occlusion or expansion of the lumen, which directly affects the blood supply of important organs such as brain, heart, kidney, etc., when serious causes the viscera function not to be complete.^[3]^ TAK usually has an occult onset, and its clinical manifestations vary greatly depending on the location of the involved vessels.

Hyperthyroidism is a common disease of endocrine system. The prevalence of hyperthyroidism is influenced by age, sex, race, thyroid function test indexes, test methods, diagnostic criteria, iodine nutritional status and other factors. The prevalence of hyperthyroidism and Graves’ disease (GD) in women is mostly in the age of 30 to 60 years old, and the prevalence of hyperthyroidism and GD in women is significantly decreased after 60 years old.^[4]^ The Thyroid hormone affects almost every cell in the body, untreated or even subclinical hyperthyroidism can lead to an increased risk of atrial fibrillation, stroke, and other cardiovascular events, as well as osteoporosis and fractures.^[5–7]^

Few cases of TAK with hyperthyroidism have been reported in young women, and in this case we report a significant improvement in blood pressure (BP) after treatment for renal artery stenosis (TAK) and hyperthyroidism.

## 2. Case presentation

A 18-year-old female with palpitation and weakness after activity. The above symptoms occur intermittently. Local hospital, BP 162/80 mm Hg. Max BP 190/87 mm Hg. Young, high BP, in order to find out the reason to our hospital. Physical examination: pulse 111 beats/min, her BP was 150/72 and 81/48 mm Hg in her right arm and left arm respectively. The thyroid gland is full, vascular bruits were heard over the left supra- and subclavian areas, neck and 2 cm above the navel. Her left upper limb pulse decreased and the rest is normal.

Right upper limb ambulatory BP: 24-hour mean BP 154/69 mm Hg, Heart rate 123 times per minute. Day time mean BP 156/70 mm Hg, Heart rate 124 times per minute. Night mean BP 147/66 mm Hg, Heart rate 119 times per minute. BP of extremities and ankle-brachial index (ABI): Left upper limb BP 94/56 mm Hg, right upper limb BP 150/67 mm Hg, left lower limb BP 75/51, right lower limb BP 76/54 mm Hg, ABI right is 0.51, left is 0.50.

**Figure 1.**
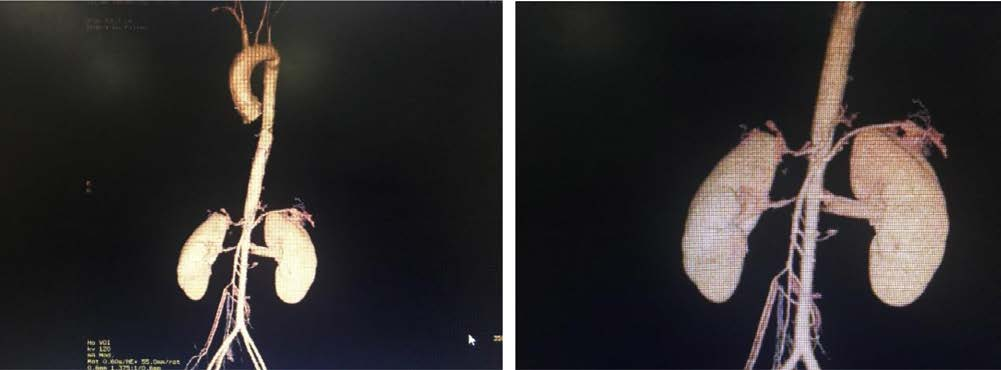
Aortic arch, thoracic aorta, abdominal aorta, left subclavian artery wall thickened, mild stenosis, right renal artery proximal severe stenosis (CTA). CTA = computer tomography angiography.

**Figure 2.**
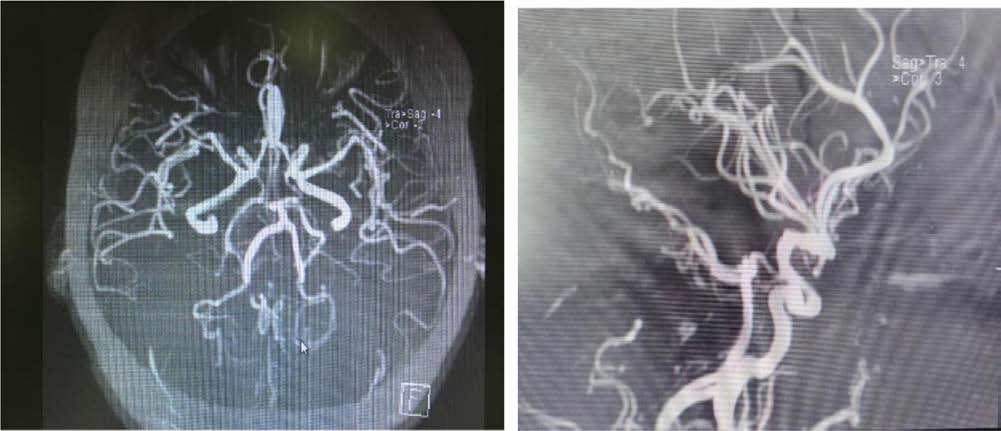
MRA of cerebral vessels. MRA = magnetic resonance angiography.

Laboratory tests showed hemoglobin 99 g/L (normal range 115–150 g/L), hematocrit 28.9% (normal range 35%–45%), mean erythrocyte volume 65 fL (normal range 82–100 fL), Mean corpuscular hemoglobin 22.3 pg (normal range 27–34 pg), C-reactive protein (CRP) 17.11 mg/L (normal range 0–10 mg/L). Erythrocyte sedimentation rate (ESR) 53 mm/H (normal range 0–20 mm/H). Triiodothyronine 4.31 (normal range 0.61–1.81 ng/mL), tetraiodothyroxine 25.6 (normal range 4.5–10.9 µg/dL), serum-free triiodothyronine 30.8 (normal range 3.5–6.5 pmol/L), serum-free thyroxine 66.52 (normal range 11.5–22.7 pmol/L), Thyrotropin (TSH) 0.008 (normal range 0.51–4.94 µIU/mL), thyroid peroxidase antibody 96.56 (normal range 0–9 IU/mL), thyroid stimulating receptor antibody 18.07 (normal range < 1.75 IU/L). The following tests were all within normal limits: Liver function tests, blood, urea nitrogen, creatinine, electrolyte, Urine routine, Adrenocortical and medulla hormone, tuberculin skin test, Antinuclear antibody, autoantibody spectrum, complement, rheumatoid factor, perinuclear anti-neutrophil cytoplasmic antibodies, cytoplasmic anti-neutrophil cytoplasmic antibodies, complement factors C3 and C4, antistreptolysin O. Chest X-ray was normal, The fundus artery was normal in fundus photography. The electrocardiogram showed sinus tachycardia, Ultrasound showed large thyroid gland with abundant blood supply and Bilateral common carotid artery wall diffuse thickening. Computer tomography angiography (CTA) showed Aortic arch, thoracic aorta, abdominal aorta, left subclavian artery wall thickened, mild stenosis, right renal artery proximal severe stenosis (Fig. 1). GFR left 59.79 mL/min, right 40.5 mL/min. Magnetic resonance angiography showed Abnormal changes in the circle of Willis of cerebral artery and M1 segment of bilateral middle cerebral artery are consistent with arteritis (Fig. 2) Thyroid nuclide imaging showed diffuse enlargement of thyroid gland with increased uptake function. Based on the results of these tests, Tuberculosis, rheumatism and other diseases were excluded. Establishing a diagnosis of second hypertension, TA and hyperthyroidism.

She was given Low salt low fat no-iodine diet, Nifedipine controlled release tablets 30 mg qd, prednisolone 1 mg/kg/day along with aspirin 100 mg, omeprazole, folic acid, calcium, and vitamin D. Methimazole, Atenolol, Leucogen, Polysaccharide iron complex. ESR, CRP reduced to normal and stable, interventional therapy was performed, According to the angiographic results, The Left subclavian artery, left axillary artery, right renal artery, left superficial femoral, artery, Right superficial femoral artery were treated with balloon dilatation. After treatment, ABI improved and The BP difference between left and right upper extremities decreased significantly. Left upper limb BP 128/72 mm Hg, right upper limb BP 148/79 mm Hg, left lower limb BP 135/71, right lower limb BP 125/69 mm Hg, ABI right is 0.84, left is 0.91. Blood pressure was lower than before, and the antihypertensive medication was adjusted to amlodipine 5 mg qd. After activity, her palpitation and weakness improved.

The BP, Blood routinet, Thyroid hormone, ESR, and CRP were used for clinical follow-up (every 3 months). ESR, CRP were within the normal range, and prednisone dosage was gradually reduced to 15 mg per day. According to the condition of thyroid function, methimazole from the beginning of 10 mg bid to 2.5 mg qd now (1 year later). Hemoglobin is in the normal range, Discontinue use of Polysaccharide iron complex.

**Figure 3.**
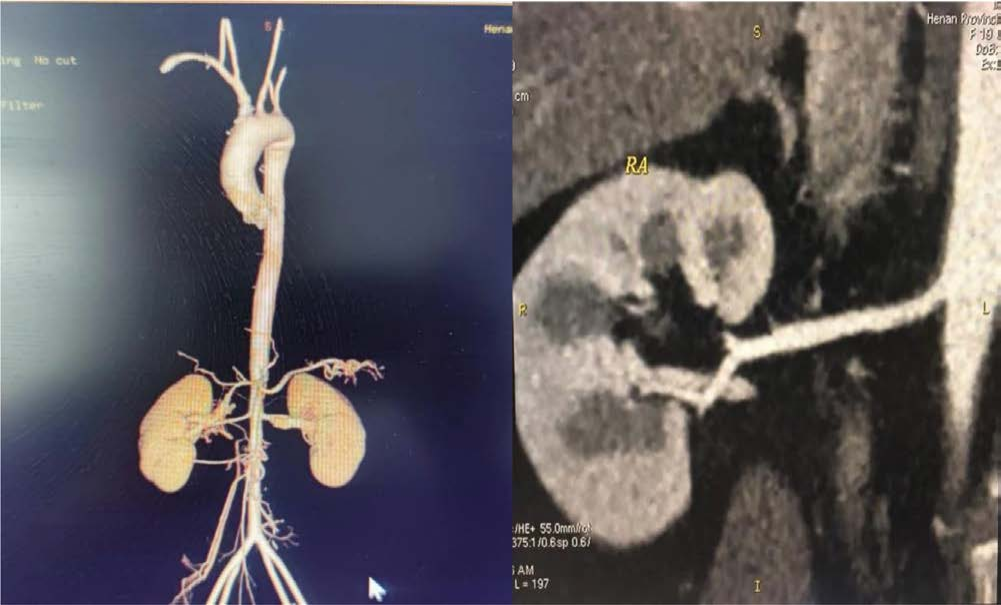
CTA was reexamined 1 yr later, no obvious stenosis. CTA = computer tomography angiography.

Renal artery CTA was reexamined 1 year later, and there was no obvious stenosis (Fig. 3). The patient is still following up in our outpatient clinic for continuous evaluation and dosage adjustment.

## 3. Discussion

TAK is a chronic inflammatory disease with an unknown cause. Current studies have shown that TAK may be related to genetic, infection, environmental and other factors.^[8,9]^ Infection may be the most important trigger of TAK. It was thought that the infection of *Mycobacterium tuberculosis, Treponema pallidum* and *Streptococcus pneumoniae* were related to TAK. Among them, the epidemiological data and pathological characteristics of Mycobacterium tuberculosis and TAK were the most close. Although the association between environmental factors and vasculitis is not well understood, there are significant regional differences in the occurrence of most vasculitis. TAK, for example, is most common among Asian women of reproductive age. Recent studies have confirmed the potential role of human leukocyte antigen in its pathogenesis.^[10]^ IL-6, which is shown to be expressed in TA arterial lesions, influences the function of many types of cells and has a role in vascular inflammation.^[11,12]^ The preliminary studies and case reports suggested that tocilizumab, a humanized anti-IL-6 receptor antibody, may be an option for refractory TA patients.^[13–16]^

There were cases of stroke, myocardial infarction and syncope in the past, and some cases of nonspecific symptoms such as ocular symptoms, joint symptoms and weakness. This case was a young female who sought medical treatment due to high BP. The symptoms of ischemia were not obvious and the limbs were weak after activity. It may be related to its age and compensation. Combined with the systemic vascular condition after examination, the course of the disease should not be short. Has involved the cerebrovascular, active anti-inflammatory treatment, inflammation in a stable period, to prevent cerebrovascular accidents. After balloon dilation of renal artery and lower limb blood vessels, BP was well controlled, ABI was significantly improved, BP systolic difference in both upper limbs was reduced from around 60 to 20 mm Hg, and symptoms were improved.

DSA is the gold standard for diagnosing TAK. However, due to its invasive, radioactive, contrast agent toxicity and limited value to vascular walls. In recent years, it has been gradually replaced by MRI angiography, CT angiography, PET-CT, color ultrasound and so on. Cases of arteritis diagnosed by MRI, ultrasound, and PET-CT have been reported.^[17,18]^ In this case, color doppler ultrasonography of carotid artery, CTA of aorta and Magnetic resonance angiography of skull all considered the inflammatory changes of arteries. It involves many blood vessels of the whole body, including cerebral vessels, trunk of the head and arm, subclavian artery, aorta, lower limb vessels, etc. It belongs to extensive TAK.

Anemia was found to be a common complication of TAK, especially in young women. In this case, the cause of anemia was also hyperthyroidism. Hyperthyroidism is a condition in which Thyroid hormone are overproduced and secreted. The prevalence of clinical hyperthyroidism, subclinical hyperthyroidism and GD was 0.78%, 0.44%, and 0.53%, respectively, in 78,470 subjects from 31 provinces of China.^[4]^ GD is one of the most common autoimmune thyroid disease in the hyperthyroidism, induce humoral and cellular immune dysfunction leading to goiter, hyperthyroidism clinical syndrome. Its pathogenic factors are mainly genetic factors, environmental factors, autoimmune 3 types. Studies at home and abroad have proved that GD is caused by the abnormality of autoimmune mechanism controlled by genetic factors. The pathogenesis of autoimmune disease is not clear. The major thyroid autoantigens are thyroglobulin, thyroid stimulating hormone receptor and TPO. The major autoantibodies are TGAB, thyroid stimulating receptor antibody and thyroid peroxidase antibody. These antigens and antibodies lymphocyte autoimmune reactions with the involvement of cytotoxic t-associated antigen (CTL) −4, interleukin (IL), Tumor necrosis factors (TNF) −N, chemokines and adhesion molecules.^[19,20]^

In 1980, a case of 32-year-old male hyperthyroidism complicated with TAK was reported. It was mentioned in the paper that 14 cases of great arteritis complicated with autoimmune thyroid disease were reported between 1962 and 1978, all female patients aged 20 to 54 years.^[21]^ Two cases of female hyperthyroidism with Crohn disease and aorta were reported in 2003. The association of these 3 diseases may not be fortuitous, possibly explained by genetic predisposing factors and disease-related iodine deficiency both involving Nuclear Factor KB pathway.^[22]^ In this case, hyperthyroidism, TAK was controlled, anemia, palpitation, and so on were significantly improved. Blood pressure is well controlled with a small dose of CCB drugs.

**Figure 4.**
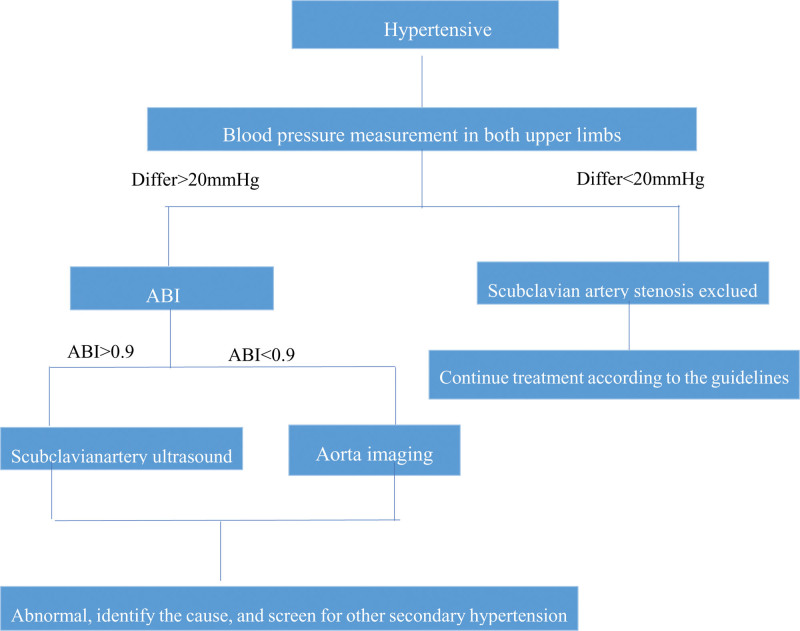
Diagnostic path.

This patient has renal artery stenosis (TAK) and hyperthyroidism, both of which are causes of secondary hypertension. Early diagnosis and treatment of TAK and hyperthyroidism can prevent serious complications. The etiology diagnosis of hypertension is very important, and it is recommended to measure both upper limbs BP in newly diagnosed patients. If BP is not measured in both upper limbs, it is likely to be missed. The cause of vascular stenosis needs to be identified, otherwise interventional treatment may lead to aggravation of the condition. If the cause of the patient is not investigated, there is no anti-inflammatory treatment, direct interventional surgery may lead to vascular occlusion (Fig. 4). When the above 2 diseases are under control, BP is easier to be controlled within the normal range, reducing the damage of target organs and complications caused by hypertension. Both of these 2 diseases are associated with autoimmunity, and there are not many cases in the past. There may be common markers of autoimmune diseases between the 2 diseases.

## Author contributions

**Investigation:** Wen-yong Dong, Xiao-lin Sun.

**Methodology:** Xiao-lin Sun.

**Project administration:** Min Liu.

**Writing – original draft:** Lian-Man He.

**Writing – review & editing:** Lian-Man He.

## References

[1] Rim Oldi SF Scherrer U Messerli FH. Secondary arterial hypertension: when who, and how to screen? Eur Heart J. 2014;35:1245–54. 24366917 10.1093/eurheartj/eht534

[2] Gornik HL Creager MA. Aortitis. Circulation. 2008;117:3039–51. 18541754 10.1161/CIRCULATIONAHA.107.760686 PMC2759760

[3] Subramanyan R Joy J Balakrishnan KG. Natural history of aortoarteritis (Takayasu’s disease). Circulation. 1989;80:429–37. 2569946 10.1161/01.cir.80.3.429

[4] Li Y Teng D Ba J. Efficacy and safety of long-term universal salt iodization on thyroid disorders: epidemiological evidence from 31 provinces of mainland China. Thyroid. 2020;30:568–79. 32075540 10.1089/thy.2019.0067

[5] Friberg L Rosenqvist M Lip GY. Evaluation of risk stratification schemes for ischaemic stroke and bleeding in 182678 patients with atrial fibrillation: the Swedish Atrial Fibrillation cohort study. Eur Heart J. 2012;33:1500–10. 22246443 10.1093/eurheartj/ehr488

[6] Baumgartner C da Costa BR Collet TH. Thyroid function within the normal range, subclinical hypothyroidism, and the risk of atrial fibrillation. Circulation. 2017;136:2100–16. 29061566 10.1161/CIRCULATIONAHA.117.028753 PMC5705446

[7] De Leo S Lee SY Braverman LE. Hyperthyroidism. Lancet (London). 2016;388:906–18. 10.1016/S0140-6736(16)00278-6 PMC5014602 27038492

[8] Johnston SL Lock RJ Gompels MM. Takayasu arteritis: a review. J Clin Pathol. 2002;55:481–6. 12101189 10.1136/jcp.55.7.481 PMC1769710

[9] Noris M. Pathogenesis of Takayasu’s arteritis. J Nephrol. 2001;14:506–13. 11783607

[10] Kimura A Ota M Katsuyama Y. Mapping of the HLA-linked genes controlling the Susceptibility to Takayasu’s arteritis. Int J Cardiol. 2000;75:S105–110. 10980346 10.1016/s0167-5273(00)00178-9

[11] Arnaud L Haroche J Mathian A. Pathogenesis of Takayasu’s arteritis: a 2011 update. Autoimmun Rev. 2011;11:61–7. 21855656 10.1016/j.autrev.2011.08.001

[12] Park MC Lee SW Park YB. Serum cytokine profiles and their correlations with disease activity in Takayasu’s arteritis. Rheumatol (Oxford, England). 2006;45:545–8. 10.1093/rheumatology/kei266 16352633

[13] Salvarani C Magnani L Catanoso M. Tocilizumab: a novel therapy for patients with large-vessel vasculitis. Rheumatology (Oxford, England). 2012;51:151–6. 22075063 10.1093/rheumatology/ker296

[14] Abisror N Mekinian A Lavigne C. Tocilizumab in refractory Takayasu arteritis: a case series and updated literature review. Autoimmun Rev. 2013;12:1143–9. 23820042 10.1016/j.autrev.2013.06.019

[15] Goel R Danda D Kumar S. Rapid control of disease activity by tocilizumab in 10 “difficult-to-treat” cases of Takayasu arteritis. Int J Rheumatic Diseases. 2013;16:754–61. 10.1111/1756-185X.12220 24382284

[16] Canas CA Canas F Izquierdo JH. Efficacy and safety of anti-interleukin 6 receptor monoclonal antibody (tocilizumab) in Colombian patients with Takayasu arteritis. J Clin Rheumatol. 2014;20:125–9. 24662551 10.1097/RHU.0000000000000098

[17] Barra L Kanji T Malette J. Imaging modalities for the diagnosis and disease activity assessment of Takayasu′s arteritis: a systematic review and meta-analysis. Autoimmun Rev. 2018;17:175–87. 29313811 10.1016/j.autrev.2017.11.021

[18] Dedushi K Hyseni F Musa J. MRI diagnosis of Takayasu arteritis in a young women. Radiol Case Rep. 2021;16:3915–9. 34703518 10.1016/j.radcr.2021.09.030 PMC8526493

[19] Ross DS Burch HB Cooper DS. 2016 American thyroid association guidelines for diagnosis and management of hyperthyroidism and other causes of thyrotoxicosis. Thyroid. 2016;26:1343–421. 27521067 10.1089/thy.2016.0229

[20] Poppe K Bisschop P Fugazzola L. 2021 European thyroid association guideline thyroid disorders prior to and during assisted reproduction. Eur Thyroid J. 2021;9:281–95. 33718252 10.1159/000512790 PMC7923920

[21] Yoshihiro S Masaro K Shunjiro K. Aortitis syndrome and hyperthyroidism-A case report and review of the Japanese literature. Kurume Med J. 1980;27:113–7. 6107399 10.2739/kurumemedj.27.113

[22] Kettaneh A Prevot S Biaggi A. Hyperthyroidism in two patients with crohn disease and Takayasu arteritis. Scand J Gastroenterol. 2003;8:901–3. 10.1080/00365520310003912 12940446

